# PR-Set7 is Degraded in a Conditional *Cul4A* Transgenic Mouse Model of Lung Cancer

**DOI:** 10.3779/j.issn.1009-3419.2015.06.15

**Published:** 2015-06-20

**Authors:** Yang WANG, Zhidong XU, Jian-Hua MAO, David. HSIEH, Alfred AU, David M. JABLONS, Hui LI, Liang YOU

**Affiliations:** 1 Thoracic Surgery Department, Beijing Chao-Yang Hospital, Capital University of Medical Science, Beijing 100020, China; 2 Toracic Oncology Laboratory, Department of Surgery, Comprehensive Cancer Center, University of California, San Francisco, CA 94143, USA; 3 Life Sciences Division, Lawrence Berkeley National Laboratory, University of California, Berkeley, CA 94720, USA; 4 Division of Diagnostic Pathology, Comprehensive Cancer Center, University of California, San Francisco, CA 94143, USA

**Keywords:** Lung neoplasms, Cul4A, AdenoCre, Mouse model, PR-Set7, γ-tubulin, Pericentrin, Cell cycle

## Abstract

**Background and objective:**

Maintenance of genomic integrity is essential to ensure normal organismal development and to prevent diseases such as cancer. PR-Set7 (also known as Set8) is a cell cycle regulated enzyme that catalyses monomethylation of histone 4 at Lys20 (H4K20me1) to promote chromosome condensation and prevent DNA damage. Recent studies show that CRL4CDT2-mediated ubiquitylation of PR-Set7 leads to its degradation during S phase and afer DNA damage. Tis might occur to ensure appropriate changes in chromosome structure during the cell cycle or to preserve genome integrity afer DNA damage.

**Methods:**

We developed a new model of lung tumor development in mice harboring a conditionally expressed allele of Cul4A. We have therefore used a mouse model to demonstrate for the frst time that Cul4A is oncogenic *in vivo*. With this model, staining of PR-Set7 in the preneoplastic and tumor lesions in AdenoCre-induced mouse lungs was performed. Meanwhile we identifed higher protein level changes of γ-tubulin and pericentrin by IHC.

**Results:**

Te level of PR-Set7 down-regulated in the preneoplastic and adenocarcinomous lesions following over-expression of Cul4A. We also identifed higher levels of the proteins pericentrin and γ-tubulin in Cul4A mouse lungs induced by AdenoCre.

**Conclusion:**

PR-Set7 is a direct target of Cul4A for degradation and involved in the formation of lung tumors in the conditional Cul4A transgenic mouse model.

## Introduction

Lung cancer is the leading cause of cancer deaths worldwide^[1]^. The central role of CRL4Cdt2 in regulating the expression of various proteins that regulate the cell cycle, and its role in determining the integrity of DNA replication and repair, suggest that various components of CRL4Cdt2 could be involved in tumorigenesis^[2]^. In mammals, the Cul4 family has another member, Cul4B, which shares 89% sequence homology and some functional redundancy with Cul4A^[3-5]^.

The importance of cell cycle mediators in human carcinogenesis is now well established^[6]^. We already showed that Cul4A is oncogenic *in vivo* and that over-expression of Cul4A leads to tumorigenesis in the mouse lung^[7]^. However, the relationship between Cul4A induced lung tumor occurrence and cell cycle remains to be determined. In our experiments, we used Cre-recombinase-induced overexpression of the *Cul4A* gene in transgenic mice to explore relationship between the *Cul4A* gene and DNA re-replication and genomic instability. Meanwhile, we will also investigate the effect of PR-Set7, the key regulator of genomic stability in mouse lung tumors. Finally, we will validate elevated protein levels of pericentrin and γ-tubulin in mouse lungs with Cul4A over-expression, as elevated levels of these proteins often indicate an increase in chromosome number, an event associated with human lung carcinogenesis.

## Materials and methods

### AdenoCre induction in Cul4A transgenic mice

Mice were infected with AdenoCre or Ade-GFP at 6-10 weeks of age. The study was approved by the University of California at San Francisco (UCSF) Institutional Animal Care and Use Committee. The intranasal administration of the adenoviruses was performed as previously described^[8]^. The AdenoCre (Ad5CMVCre) and Ade-GFP (Ad5CMVeGFP) were purchased from the Gene Transfer Vector Core of the University of Iowa, and have been used in several studies^[9]^. To generate a mouse model that would conditionally over-express Cul4A protein, we used the pCALL2 vector^[10]^. Transgenic mice were anesthetized with 2.5% Avertin via intraperitoneal injection, after which half of the mice inhaled approximately 10^6^ or 10^7^ particles of AdenoCre introduced directly into the lungs. The other half inhaled Ade-GFP without AdenoCre. Eight weeks later, the mice were killed, and the lungs were dissected and sections analyzed.

### Histological analysis and immunohistochemistry

Animals were killed at the post-infection times indicated and subjected to full necropsy for histological and immunohistochemical analyses. Sections were blocked with 4% normal goat serum in PBS with 0.2% Triton for 2 h at RT before incubating overnight at 4 ℃ with the properly diluted antibodies: anti-PRSet7 1:100 (2996, CellSignaling); anti-γ tubulin 1:5, 000 (ab11317, Abcam); anti-pericentrin 1:2, 000 (ab4448, Abcam).

### Immunohistochemical analyses and statistical analysis

Correlation analyses were performed using SPSS 13.0 (SPSS Inc, Chicago, IL, USA). We used 0, 1, 2, and 3 as representatives of -, +, ++, and +++, respectively. The following scoring system was employed: -, no stain; +, weak staining (10% or above stained cellularity considered as positive); ++, moderate staining (30% or above stained cellularity considered as positive); +++, strong staining (positive). All scoring systems were under low magnification (×10).

## Results

### The overt adenocarcinomas (AD) were present since 20 wk post-infection with AdenoCre

Infected Lox-Stop-Lox Cul4A mice (hereafter referred to as Lox-Cul4A) were killed at 8, 12, 16, 20, 24 and 24 wk post-infection. At 20 wk post-infection with AdenoCre, the surface of Lox-Cul4A lungs had a cobblestone appearance, and histological sections showing a single lesion. At 24 wk post-infection with AdenoCre, visible tumors were clearly seen on the surface of the Lox-Cul4A mouse lungs, with numerous severe isolated adenocarcinoma-like lesions visible under a microscope (Fig 1).

**1:**
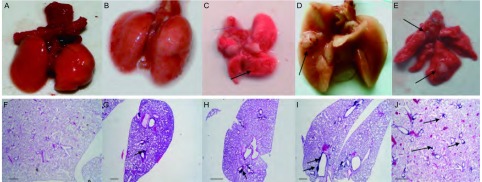
Tumor pathology in Cul4A over-expression mouse lungs. A: Wild-type mouse lungs 8 wk post-infection with Ade-GFP. The surface of the lungs is smooth and uniform. B: Lox-Cul4A mouse lungs 12 wk post-infection with AdenoCre. The surface of the lungs is also smooth and uniform. C: Lox-Cul4A mouse lungs 16 wk post-infection with AdenoCre. The surface of the lungs has a bumpy appearance, arrows indicate the lesions. D: Lox-Cul4A mouse lungs 20 wk post-infection with AdenoCre. An isolated cobblestone-like nodule on the surface of the lungs is visible; the arrow indicates the lesion. E: Lox-Cul4A mouse lungs 24 wk post-infection with AdenoCre. Several nodules on the surface of the lungs have appeared; arrows indicate the lesions. F: Histological sections of wild-type mouse lungs 8 wk post-infection with AdenoCre (HE). G: Histological sections of Lox-Cul4A mouse lungs 12 wk post-infection with AdenoCre; the arrow shows a single lesion (HE). H: Histological sections of Lox-Cul4A mouse lungs 16 wk post-infection with AdenoCre; arrows indicate isolated lesions (HE). I: Histological sections of Lox-Cul4A mouse lungs 20 wk post-infection with AdenoCre; arrow shows a single adenocarcinoma-like lesion (HE). J: Histological sections of Lox-Cul4A mouse lungs 24 wk post-infection with AdenoCre; arrows show diffuse severe adenocarcinoma-like lesions (HE). Scale bar indicates 200 *μ*m.

### Cul4A promotes PR-Set7 degradation: immunohistochemical analysis

Stainings of PR-Set7 in mouse lung sections induced with Ade-GFP were weakly to moderately positive across all time points (Fig 2A, C, E); whereas stainings of PR-Set7 in the preneoplastic and tumor lesions in AdenoCre-induced mouse lungs was either weakly positive or else negative across all time points (Fig 2B, D, F).

**2:**
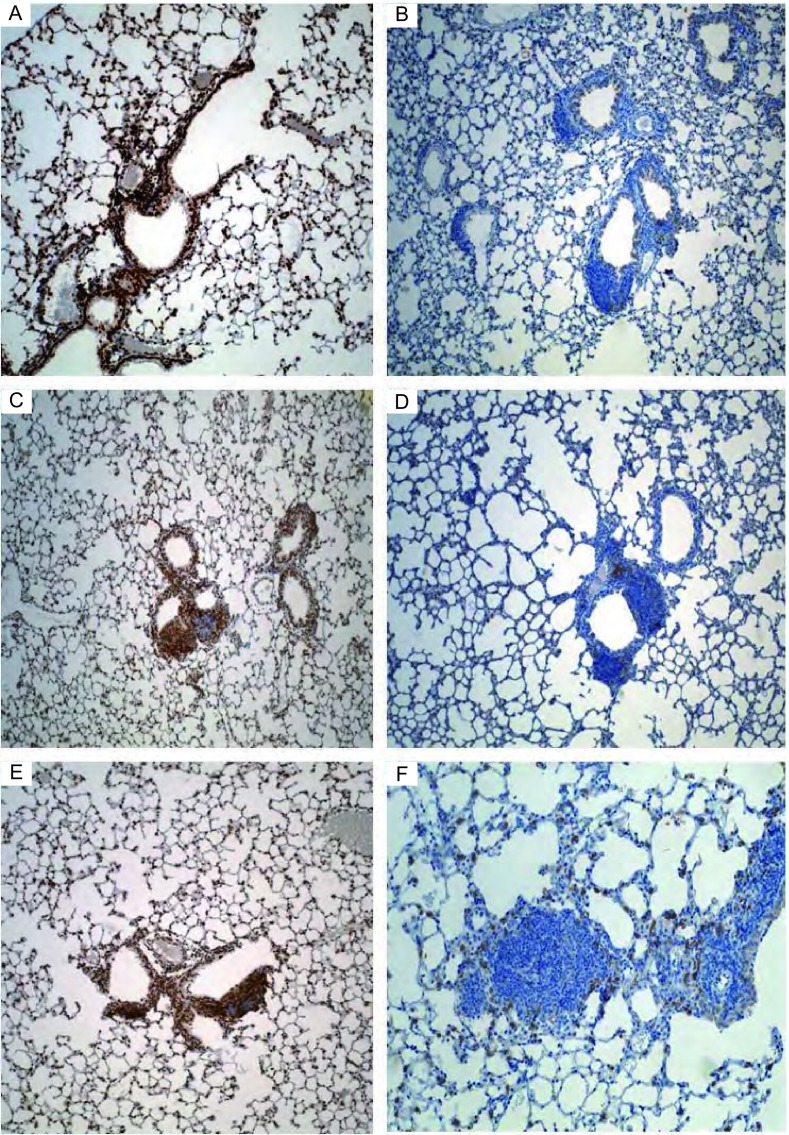
Immunostainings of PR-Set 7 in the mouse lung sections at the indicated time. A: 8 wk with Ade-GFP (SP). B: 8 wk with AdenoCre (SP). C: 12 wk with Ade-GFP (SP). D: 12 wk with AdenoCre (SP). E: 16 wk with Ade-GFP (SP). F: 16 wk with AdenoCre (SP).

### Up-regulation of γ-tubulin and pericentrin were found in Lox-Cul4A mouse lungs induced by AdenoCre

The protein level change of γ-tubulin and pericentrin in Cul4A mouse lungs induced by AdenoCre were both higher than those of mouse lungs induced with Ade-GFP (Fig 3).

**3:**
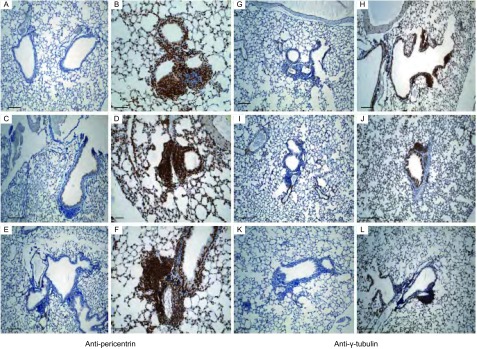
Immunostainings of pericentrin and γ-tubulin in mouse lung sections. A: 8 wk with Ade-GFP (SP). B: 8 wk with AdenoCre (SP). C: 12 wk with Ade-GFP (SP). D: 12 wk with AdenoCre (SP). E: 16 wk with Ade-GFP (SP). F: 16 wk with AdenoCre (SP). G: 8 wk with Ade-GFP (SP). H: 8 wk with AdenoCre (SP). I: 12 wk with Ade-GFP (SP). J: 12 wk with AdenoCre (SP). K: 16 wk with Ade-GFP (SP). L: 16 wk with AdenoCre (SP). Scale bar, 100 *μ*m.

## Discussion

To determine whether over-expression of Cul4A results in degradation of PR-Set7 expression, we compared the PR-Set7 expression in lung tumors and control lungs by using IHC analysis. To maintain genome integrity, DNA replication must be tightly regulated so that replication only initiates once at each replication origin during S phase. Here, we investigated the expression of PR-Set7 following the gain-of-function of Cul4A in the progression of mouse lung tumor. Staining of PR-Set7 in mouse terminal bronchiole epithelial cells induced with Ade-GFP were between weak moderate positive (++) and strong positive (+++) across all time points. Staining of Set8 in the preinvasive and adenocarcinomous lesions in AdenoCre-induced mouse lungs was weak positive (+) or negative (-) across all time points. These results gave rise to the hypothesis that the initiation and progression of mouse lung tumor elicited by over-expression of Cul4A was through degradation of PR-Set7. Similar to CDT1 (data not shown), with this mouse model, we identified that Cul4A as the principal E3 ubiquitin ligase responsible for PR-Set7 proteolytic degradation. Interestingly, a recent study also determined that Set8 (PR-Set7) ubiquitination by SCFSkp2, and subsequent degradation of PR-Set7 at G_1_, were associated with S-phase entry^[11]^. Lack of PR-Set7, as the methyltransferase that monomethylates histone H4 on lysine 20 (H4K20me1)^[12]^, results in massive DNA damage during S phase and improper chromosome condensation in mitosis^[13, 14]^. During the cell cycle, PR-Set7 is most abundant during G_2_ and M phases, and low during S phase^[15]^. Concomitant with its increase in the G_2_ and M phases, PR-Set7 promotes a transient accumulation of H4K20me1^[13]^. H4K20me1, which promotes chromatin condensation, may be necessary for proper mitosis, and may also impact the subsequent S phase. As our results showed that PR-Set7 is degraded in response to over-expression of Cul4A, we propose that Cul4A-mediated degradation of PR-Set7 in the preinvasive lesions in mouse lung sections induced might prevent H4K20me1 accumulation during S phase, thereby preventing premature chromatin condensation. We believe that this interferes with genome duplication, induces DNA damage, and activates the p53 tumor suppressor pathway (data not shown). Importantly, these reports demonstrate that inappropriate levels of PR-Set7 in this mouse model could result in profound cell cycle defects including the inability to initiate S phase, the re-replication of DNA, and the improper timing of mitotic progression. The replication-coupled down-regulation of PR-Set7 is a critical mechanism that defines the functional window of PR-Set7 during the cell cycle, contributing to the orderly execution of DNA replication and mitosis.

Here we described an efficient method for evaluation of the centrosome amplification by quantifying the protein levels of γ-tubulin and pericentrin, two constitutive centrosomal protein. The function of γ-tubulin was clearly determined by purification of the tubulin ring complex (Gamma-TuRC) that revealed a ring shape and a substructure able to nucleate microtubule polymerization *in vitro*^[16]^. Increased accumulation of microtubules, observed in reconstructed eggs, may also play a role in centrosome dysfunctions by causing abnormal microtubule organization and aneuploidy^[17]^. It is also reported that the prognosis of bladder cancer is significantly worse with centrosome amplification than without. Thus, evaluation of centrosome abnormality could be used as a prognostic biomarker for the progression and recurrence of cancer^[18]^. Various types of genetic lesions can be found in cancer cells, including DNA sequence mutations (*i*.*e*. point mutation, insertion and deletion, recombination, *etc*.) and chromosome mutations [*i*.*e*. translocation, double-minute chromosomes, aneuploidy (chromosome loss and gain), *etc*.]^[19]^. Although it is apparently meaningless for us to label which type of mutation is most important for carcinogenesis in this mouse model, it is clear that aneuploidy influences the rate of this mouse model tumor progression to a great extent, since gain of even a single chromosome can introduce multiple mutations required for acquisition of malignant phenotypes. We were unable to determine the causes for centrosome number increases in the mouse model, but loss of cell cycle regulators such as p21 (data not shown) may be related to supernumerary centrosomes.

In this paper, we further validated that key regulator of genome stability, PR-Set7 is involved in the development of mouse lung tumors, and found high protein levels of pericentrin and γ-tubulin in mouse lung tumors. Future studies are required to test this possibility and others to clarify the cellular and molecular underpinnings of the disorders associated with elevated levels of pericentrin and γ-tubulin in human lung cancers. A better understanding of Cul4A signaling pathways and mechanisms and their effects on the growth of lung tumors in our mouse model could provide new insight, thus contributing to future experimental therapies.

## Acknowledgements

We are grateful for support from the Kazan, McClain, Abrams, Fernandez, Lyons, Greenwood, Harley & Oberman Foundation, Inc; the Estate of Robert Griffiths; the Jeffrey and Karen Peterson Family Foundation; Paul and Michelle Zygielbaum; the Estate of Norman Mancini; and the Barbara Isackson Lung Cancer Research Fund. We thank Lorretta Chan in the UCSF Cancer Center Tissue Core for her help. We also thank Pamela Derish in the UCSF Department of Surgery for editorial assistance with the manuscript.
